# The Development of a UK Culturally Adapted and Modified Version of the Person Attuned Musical Interactions Manual: Protocol for a 2-Phase Mixed Methods Study

**DOI:** 10.2196/43408

**Published:** 2023-04-18

**Authors:** Bryony Waters, Martin Orrell, Orii McDermott

**Affiliations:** 1 Institute of Mental Health School of Medicine University of Nottingham Nottingham United Kingdom

**Keywords:** psychosocial intervention, music, dementia, interactions, care homes, care staff training tool, tool, research, impairment, training, communication, intervention, quality of life, care, development, language

## Abstract

**Background:**

Previous research has suggested that care home interactions need significant improvements, especially those between staff and residents with dementia. Reasons for the lack of interactions are staff time pressures and residents’ language impairments. Although residents may experience reduced language abilities, they can continue to communicate through other forms, including nonverbal communication and music. Person Attuned Musical Interactions (PAMI) is a staff training tool that provides staff with music therapy skill-sharing to promote high-quality interactions between staff and residents using nonverbal communication and music. The tool was originally developed in Denmark. To ensure that the tool is appropriate for UK care homes, a team of researchers in the United Kingdom have modified and culturally adapted the tool.

**Objective:**

This study aims to investigate the appropriateness of the adapted and modified manual for UK care homes and to explore the impact of PAMI on residents with dementia and care staff.

**Methods:**

The project consists of 2 phases, a qualitative field-testing study and a mixed methods evaluation study, which have been developed following the Medical Research Council’s guidelines for complex interventions. Care staff and residents with dementia will be recruited from care homes in Lincolnshire, where the care staff will be trained in the PAMI intervention before implementing the intervention in their daily routines. Fortnightly reflective sessions will be provided throughout the phases to provide supervision and monitoring. The qualitative methods include interviews, reflective session transcripts, diary entries, and resident experience questionnaires. The quantitative outcome measures are residents’ music engagement, staff’s dementia competence, residents’ quality of life, and staff burden. The resident’s music engagement will be administered at 9 fortnightly time points. Staff’s dementia competence, resident’s quality of life, and staff burden will be administered at preintervention and postintervention time points.

**Results:**

The study has been funded by The Music Therapy Charity as part of a PhD studentship. The study began recruiting in September 2021. The research team aims to publish the results of the first phase in July to September 2023 and those of the second phase in October to December 2023.

**Conclusions:**

This study will be the first to investigate the modified version of PAMI. Therefore, it will provide feedback on the appropriateness of the manual for UK care homes. The PAMI intervention has the potential to offer high-quality music intervention training to a larger population of care homes who may currently be restricted by finances, the availability of time, and a lack of training opportunities.

**International Registered Report Identifier (IRRID):**

DERR1-10.2196/43408

## Introduction

### Care Homes and Residents With Dementia

People reside in care homes for several reasons, including old age, health conditions, and disability, but generally, care home residents require substantial help and support to complete activities of daily living, including personal care, and to access leisure activities. In the United Kingdom, between 408,371 and 490,326 individuals live in approximately 17,079 care homes [1,2]. Approximately 70% of the residents in care homes have a diagnosis of dementia [3]. Dementia is an umbrella term for a range of progressive conditions characterized by a slow deterioration in brain function, including memory, language, decision-making, and judgment [4-6]. Individuals with dementia can experience a range of symptoms because of the deterioration, including disorientation, anxiety, depression, apathy, agitation, restlessness, hallucinations, mobility issues, and personality changes, which can affect an individual’s ability to complete daily tasks and live independently.

### Care Home Communication

The care and assistance staff provide to residents compel the need for a strong relationship based on trust, respect, and understanding [7,8]. Care staff should aim to provide person-centered care and interactions to tailor care to the resident’s needs, preferences, impairments, and abilities [9-11]. Staff should consider the individual as a whole person who is treated with respect and dignity. Person-centered care can be inconsistent among staff and care homes. During a 6-hour observation period, Willemse et al [12] reported, on average, 1.5 meaningful interactions, with one-third of participants experiencing zero meaningful interaction during the observation. Care home interactions are not only infrequent but can also be short, be fragmented, sometimes consist of “Elder speak,” and be task orientated in nature [13,14]. “Elder speak” refers to interactions characterized by simplistic vocabulary and grammar, shortened sentences, slow speech, elevated pitch, and inappropriate use of endearments [14-16]. “Elder speak” has been compared with interaction techniques typically used with babies and leaves individuals feeling patronized. The frequency and quality of interactions generally correlate with the severity of the individual’s dementia and impairments, including limited verbal language [14,17]. Understaffing and time pressures have been suggested to explain the limited interactions in some care homes and result in prioritizing physical care and safety over meaningful activities and interactions [18]. However, meaningful activities and interactions are equally essential to ensure that the resident’s psychological, social, and emotional needs are met; the resident’s identity remains; and dementia symptoms are managed. Therefore, interventions and staff training targeting interactions and meaningful activities in care homes without increasing staff time or burden are vital.

### Music in Dementia Care

Although individuals can experience reduced verbal language, involvement in interactions can continue through nonverbal communication and other alternative communication styles [17,19,20]. However, as they are untraditional and less familiar to individuals, staff can miss or misinterpret more ambiguous and subtle communication cues [20].

Music, consisting of both verbal and nonverbal elements, has been highlighted as a potential alternative communication form accessible to individuals with dementia. Although language processing significantly deteriorates as dementia progress, music processing seems to remain intact, potentially owing to music processing requiring nearly all brain areas [21-23]. Music is an inclusive and adaptable tool not limited by age, culture, or health conditions and has been evidenced as a multibeneficial tool for various health conditions, including dementia [24,25]. Music in dementia care has the potential to improve well-being, alertness, anxiety, depression, agitation, and activity disturbance [26-28]. When care staff facilitate music activities, research has reported improvements in quality of life, job satisfaction, communication skills, stress, and burden [29]. Typically, music can be categorized into 2 intervention types: music therapy, facilitated by specialist music therapists [30]; and music activities, facilitated by care staff, family members, volunteers, and musicians. Both have highlighted the benefits of incorporating the intervention into dementia care. The most appropriate intervention depends on the aims of the intervention, symptoms, residents’ preferences, and care home factors such as time and finances.

In the United Kingdom, music therapists are limited, with only 200 to 250 individuals working in the dementia field [30], resulting in the intervention being restricted to a limited number of individuals. In addition, care home finances can restrict care homes from employing music therapists. Research highlights that music therapy benefits generally only remain during and immediately after the session [31]. Therefore, to maintain the benefits, regular daily doses of music interventions should be provided in care homes, which may not be plausible with music therapy. Music activities have more flexibility and can be provided by anyone, making them a more viable option for long-term use in a larger proportion of care homes. When music activities are of high quality and implemented successfully, they can provide similar benefits to music therapy [32-36]. However, the implementation of care home music activities is currently minimal. In 2017, only 5% of care homes provided good quality arts and music interventions [37]. The lack of staff time knowledge, resources, and training may explain the lack of music interventions in UK care homes [38-40]. The lack of available time has been highlighted as a potential barrier to staff interactions and music interventions. However, a series of studies investigating music therapeutic care (MTC) demonstrated the plausibility of incorporating music into personal care tasks [32,41-45]. Because music was incorporated into other tasks, staff did not have to spend additional time or bear additional burden; instead, the intervention improved task efficiency. However, the issue remains that low-quality music activities implemented unsuccessfully can be ineffective and even harmful to residents with dementia [46].

### Music Therapy Skill-Sharing

Music therapy skill-sharing provided by music therapists has been suggested as a solution to improve the implementation of high-quality music interventions facilitated by staff in care homes. Music therapists share their skills and knowledge of music therapy, allowing staff to incorporate indirect music therapy practices into their routines [46]. As previously highlighted, staff and family members may lack knowledge and experience in music interventions. Inappropriate or lack of space and support for staff to reflect on how to be with the individual and focus solely on the content can lead to a loss in benefits [40,46,47]. Currently, music therapy skill-sharing only occurs in care homes with employed music therapists. A structured manual developed from evidence-based practices that train staff in the type of music skills and how facilitators should deliver skills would enable music therapist knowledge to be shared with and accessible to a larger population of care homes.

### Person Attuned Musical Interactions Manual

Person Attuned Musical Interactions (PAMI) is a structured staff training tool developed based on previous research that provides staff with music therapy skill-sharing training. The tool was initially created by a team of music therapist researchers at Aalborg University, Denmark [48], which aimed to promote 2-way attuned music interactions between care staff and residents with dementia. In the intervention, the PAMI research team train music therapists, who then go into care homes to train staff. The training provided to care staff consists of 3 elements: group theory and reflective training, group practical supervision, and individual practical supervision. In the group theory and reflective training, staff gain insight into the theory behind PAMI, complete reflective exercises, and learn about the PAMI skills. The group and individual supervision sessions involve the music therapists working with the care staff on the care home floor to assess and guide staff on the PAMI skills by observing them using the skills with residents. All the elements of the PAMI training are completed in person. On the basis of cultural adaptation intervention research [49,50]. A modified version of PAMI was developed by a team of researchers at the University of Nottingham to ensure that PAMI was culturally appropriate for UK care homes (Waters, B, Orrell, M, and McDermott, O, unpublished data, March 2023).

The researchers followed 2 cultural adaptation frameworks, the Barrera and Castro [51] framework and the Formative Method for Adapting Psychotherapy [52], to guide the development process. The first author’s thesis reports the manual development process and the differences between the Danish and Modified version of PAMI (Waters, B, unpublished data, March 2023). Both frameworks advise that once adapted, the newly adapted intervention should be reviewed with stakeholders to test its appropriateness and obtain preliminary results.

### Aims

The aims of this study are as follows:

1. To investigate the suitability, manual layout, and clinical relevance of the PAMI-Modified (PAMI-M) manual
2. To investigate the cultural adaptation appropriateness of the PAMI-M manual
3. To identify changes in staff’s and residents’ behavior and mood after using the PAMI-M manual
4. To identify staff’s views on using the PAMI intervention within their care home
5. To investigate the factors influencing the implementation of PAMI in UK care homes

## Methods

### Medical Research Council Guidelines

**Figure 1:**
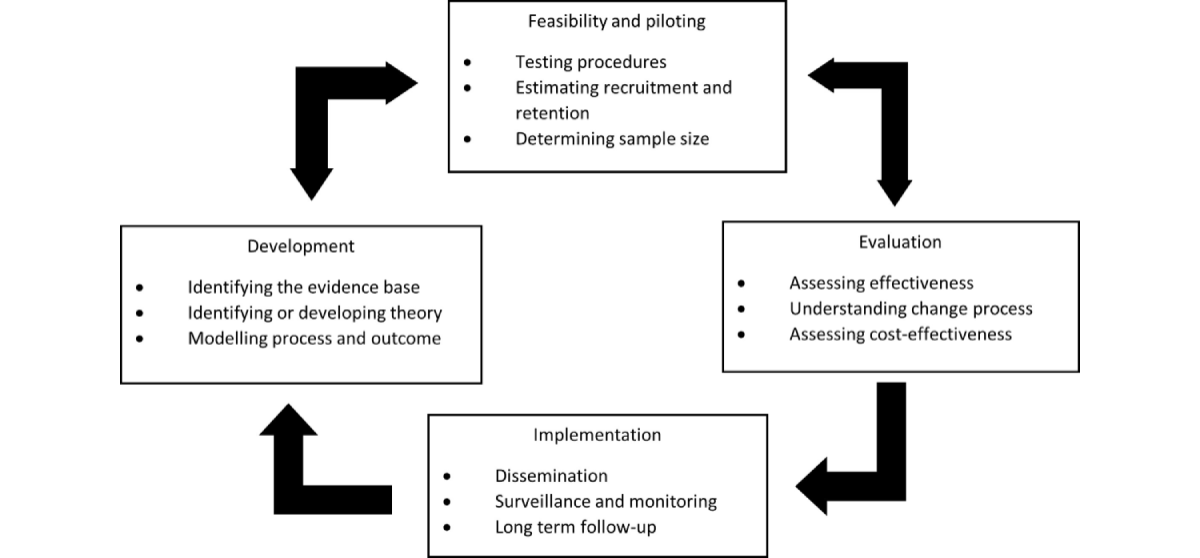
Medical Research Council complex intervention framework.

The research team identified PAMI as a complex intervention; therefore, the Medical Research Council (MRC) guidelines have been followed for this study. Complex interventions refer to interventions consisting of several interacting components. Interventions may be complex for several reasons, including the number and difficulties of behaviors of those receiving the treatment, the requirements of the facilitator, the number and variability of outcomes, the degree of flexibility, tailoring of the intervention, and the number of groups targeted. The MRC created a framework for researching complex interventions to aid researchers in designing, running, and reporting complex interventions [53]. The framework consists of 4 phases (Figure 1): development, feasibility and piloting, evaluation, and implementation. This protocol focuses on the development phase and begins to explore the feasibility and piloting phase.

### Study Overview

**Figure 2:**
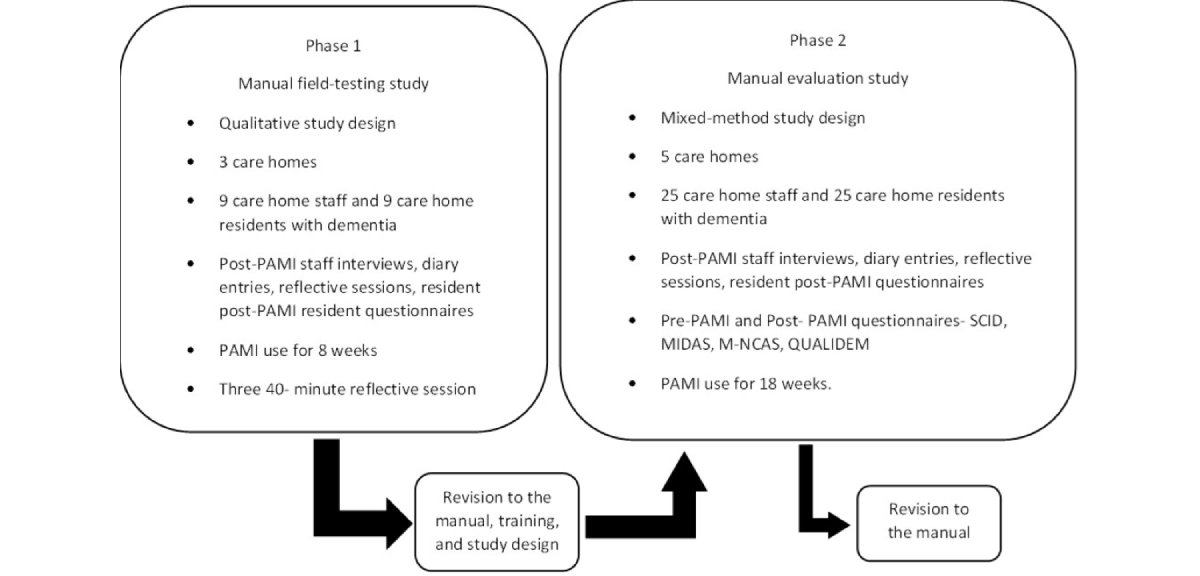
Study overview consisting of 2 phases and 2 revisions stages. The study overview was developed based on the Medical Research Council guidelines for complex interventions and culturally adapting intervention frameworks. MIDAS: Music in Dementia Assessment Scale; M-NCAS: Modified Nursing Care Assessment Scale; PAMI-M: Person Attuned Musical Interactions-Modified; SCIDS: Staff Competence in Dementia scale.

Two phases will be conducted to investigate the aims, which follow the stages of the MRC guidelines and cultural adaptation frameworks. A field-testing qualitative study will review the culturally adapted manual with its intended stakeholders to ensure appropriateness. Data will be collected on the factors influencing the implementation of the intervention and stakeholders' (care staff and residents) experience with PAMI, including changes in behavior and mood and care staff’s views on using the intervention. The data collected in the first phase will aid adaptations to the manual in preparation for the second phase. The second phase is a mixed method study that will continue reviewing the culturally adapted manual with its intended stakeholders. In addition, the study will collect preliminary data on the impact of PAMI on staff and residents to begin investigating the feasibility and piloting stage of the MRC guidelines. The data collected in the second phase will aid final manual revisions and be used to develop future studies to continue exploring the feasibility and piloting stage of the MRC guidelines. Figure 2 displays the study overview.

### Ethics Approval

The 2 phases received ethics approval from the London-Harrow Research Ethics Committee on June 29, 2021 (reference number 21/LO/0283). Amendments to the study were approved by the London-Harrow Research Ethics Committee on March 22, 2022.

### National Health Service Trust Partnership

A partnership with the Lincolnshire Partnership National Health Service (NHS) Foundation Trust was created early in the manual development process, which will assist with running the studies in care homes. As part of the partnership, PAMI will be advertised through the trust’s Enabling Research in Care Homes (ENRICH) organization. ENRICH is a national organization developed by the National Institute for Health Research (NIHR) to support care home staff, care home residents, residents’ families, and researchers to implement research into care homes more smoothly, accessibly, and effectively.

The Lincolnshire trust developed a pilot in-reach scheme in 2020 to improve the accessibility of care home research within Lincolnshire. The partnership between the research team and Lincolnshire NHS trust has led to an in-reach practitioner (RF) joining the team. The in-reach practitioner will liaise between the researchers and care homes with whom they already have a rapport. This rapport means that the in-reach practitioner is already aware of care homes potentially interested in the PAMI research, reducing the time needed for introductions, providing a recognizable face, and thus reducing anxiety among potential participants. The in-reach practitioner will support the researchers in setting up and running the study. The role involves introducing the study to care homes, recruiting participants, distributing documents, assessing capacity, obtaining consent, introducing the care homes to the research team, collecting documents, and being an in-person contact for the research team. When designing the study, the research team was uncertain about the possibility of the researchers attending the care homes owing to the COVID-19 pandemic; however, the in-reach practitioner is an NHS role, allowing them to continue attending care homes throughout the studies. The care homes involved in the in-reach pilot scheme belong to ENRICH and were invited to join the scheme.

### Recruitment

Care homes will be recruited first through the in-reach scheme or advertisement through the Lincolnshire ENRICH. Once the care homes are recruited, 2 participant types will be recruited: care staff and residents with dementia. The researchers will contact care home managers and introduce the team to interested individuals. A management introductory letter will introduce the study and provide the potential benefits to the residents, care staff, and care home. The introductory letter will also inform managers of the required time commitment from themselves, the staff, and the residents to ensure that their care home can commit the required time. The welcome pack also contains introductory letters for the care staff, residents, and residents’ families. The care home managers will be asked to distribute the welcome letters to the staff and residents. Care staff will be recruited first before residents to enable the staff to communicate the study to the residents. As the researchers cannot access residents’ care plans, the researchers require care staff to distribute the information sheets to eligible individuals. Interested individuals can contact the researchers using the provided contact details or inform the in-reach practitioner when she attends the care home of their wish to participate. Information sheets and consent forms will be provided to interested individuals, with them given a week to consider their participation. The in-reach practitioner will organize visits with the care homes during recruitment to discuss the study and answer questions. Once care staff and residents are recruited, the residents will be paired with the participating care staff they have the most direct contact with daily.

### Informed Consent

Written informed consent will be collected from all participants at the start. There are different information sheets for staff and residents. A dementia-friendly version of the resident information sheet is available for residents who may struggle to comprehend the formal participation sheet. If individuals wish to participate, a consent meeting is organized, where they can review the documents with the in-reach practitioner and sign the consent form. The consent meeting can be completed either in person at the participating care home or remotely via Microsoft Teams (Microsoft Corp). During the meeting, potential participants are given time to ask any questions or concerns. Once the consent forms are signed, 1 copy is given to the participant, and the research team keeps 1 copy. A third copy is kept in the resident’s care file when resident participants consent. Consent is an ongoing process; therefore, the research team will continue checking participants’ consent. Some resident participants may be unable to consent verbally; therefore, the researchers will work with the care home to determine the most appropriate form of communication for each resident.

### Researching Adults Who Lack Capacity

The studies involve participants who may lack capacity owing to their dementia diagnosis. The Mental Capacity Act 2005 [54] will be followed to determine participants’ capacity to consent. All research team members have previously worked in care homes, where their role required the understanding of the lack of capacity. All members have also completed training in informed consent with adults lacking capacity. With assistance from the care homes, the in-reach practitioner will assess the resident’s capacity to consent during the consent meeting, which involves assessing whether the resident understands the study and what is required of them and ensuring that they understand the consent form and can follow the instructions to sign the form. A consultee, who is a family member, a friend, or an appointed power of attorney, will be appointed for residents who are deemed to lack capacity. Once a consultee is appointed, they will be provided with an invitation letter, a consultee information form, and a consultee declaration form, with time to consider their relative’s (the term relative is used to also refers to those who’s consultee is a friend or power of attorney) participation in the study before a consultee meeting is organized. The consultee meeting can take place in person or remotely via Microsoft Teams. During the consultee meeting, the consultee will be asked to sign the declaration form if they wish their relative to take part in the study. Staff and consultees can use the dementia-friendly information sheet to discuss with residents their participation in the study. For residents who are deemed to lack capacity to consent, on top of their consultee’s declaration to participate, assent will be obtained to ensure that they agree to partake. Assent will be collected to allow residents to remain involved in the decision process when not legally able to consent. The most appropriate communication form for residents will be used to obtain assent. Researchers will ensure that residents continue to consent throughout the study, using their choose communication form. For residents unable to verbally assent, staff and researchers will assess residents’ behavior for signs of wishing to withdraw or distress throughout the study.

Dementia is a progressive condition; therefore, some resident participants could lose capacity during the study. The researchers will review the resident’s capacity throughout. If a resident is deemed to lose the capacity to consent during the study, a consultee will be appointed and asked to complete the consultee declaration form to allow the resident to continue the study.

### Withdrawal

Care staff participants will be reminded at the start of each reflective session that their participation and their resident’s participation are voluntary, and they can withdraw at any time without providing a reason. Participants will also be reminded that withdrawal from the study will not affect the care they receive as a resident or their employment as staff. During the reflective sessions, care staff will be asked to report any PAMI issues and whether residents have displayed signs of distress or wish to withdraw. In-between reflective sessions, participants can contact the researchers via email or telephone. All study activities will cease immediately if a participant withdraws, and no further data will be collected. However, the data that have already been collected will be used in the final analysis. As participants partake in the study as dyads, both participants will be withdrawn from the study. The only exception is if only the staff interview remains and the resident withdraws; in this case, the staff member can complete the interview but will only be able to report on their experience of the training. Withdrawn participants will not be replaced.

### Setting

The 2 phases will be conducted in Lincolnshire care homes caring for individuals with dementia. The care homes can be private, voluntary, NHS or council owned and provide either residential, nursing, or both types of care. All care homes must meet the eligibility criteria.

The care home eligibility criteria are as follows: they should have received a score of “Needs improvements” or “good” on their most recent Care Quality Commission report and have adequate Wi-Fi for staff to be able to attend the PAMI training and reflective session.

The in-reach practitioner will review care homes’ eligibility to determine to whom to introduce the study. The care homes in the in-reach pilot are all part of the ENRICH group, which promotes and supports care homes with research.

### Remote Delivery

When designing the study, the UK COVID-19 guidelines restricted researchers from attending care homes. As a result, several study elements have been converted to take place remotely. The training, reflective sessions, and interviews will be completed remotely using a conference call platform. Microsoft Teams was selected because of its ease of use; it allows up to 250 individuals to join a call; it allows for screen sharing; it is not time limited; it can record sessions; and it is encrypted, making it safer. The NHS Transformation Directorate Department, formally known as NHS user experience, confirmed in 2020 that Microsoft Teams is safe to use in health care [55,56]. A Microsoft Teams channel will be created for each care home where the training session, reflective sessions, and interview meetings are scheduled. A Microsoft Teams information sheet has been created, providing information on joining Microsoft Teams channels and meetings. The information sheet will also be provided to consultees who complete their consultee advice meeting remotely. For ease and consistency, the research team decided to continue running the studies remotely when the UK COVID-19 guidelines were lifted.

### Intervention

The PAMI intervention can be split into 3 elements: the paper copy of the PAMI manual, the initial training session, and fortnightly reflective sessions.

#### Manual

The PAMI manual consists of theory, reflective exercises, examples, and practical skills. The manual can be sectioned into 6 categories: interactions and music, the voice, framing, balancing, connecting, and practical tips and resources. Colored boxes have been used to separate the different elements to make it easier for staff to locate information. Staff are required to bring the paper manual to the initial PAMI training, as the reflective exercises in the manual are completed during the training.

The first section, interactions and music as communication, provides staff with theory on interacting with individuals with dementia. PAMI aims to be able to train all staff regardless their level of experience and training; therefore, this section provides the foundations of interactions in dementia care to ensure that everyone is at the same level before exploring the PAMI skills. This section also starts to explore using music therapeutically and the research highlighting the benefits of music for individuals with dementia. The second section begins to explore the voice as a musical element. The section reflects on when and how staff use their voice currently. The music parameters of the voice, including pitch, rhythm, tone, and volume, are explored in relation to the staff’s roles. Framing explores adapting the care home environment to create security and predictability. This section introduces the skills of using music as a cue for tasks and sound environments. Balancing explores recognizing and adapting residents’ arousal states using music, the voice, and the body when residents experience agitation or apathy. Staff also gain skills to recognize their arousal state and regulate it to ensure that their emotions are not impacting their interactions with residents. Connecting explores using meaningful music to connect and create relationships. Staff explore reminiscence and music therapy skills such as attunement, validation, and holding. The connecting section introduces the PAMI music care plan, providing residents with a tailored music plan that staff can use to implement music successfully. The final section provides practical tips to improve the implementation of the intervention.

#### Training Session

The primary researcher (BW) will facilitate the training sessions with assistance from a music therapist (BS). BW is a nonmusic therapist with a background in psychology and has worked as a care home well-being therapist. BW was the researcher who developed the modified PAMI manual as part of her PhD. (Waters, B, unpublished data, March 2023). The PhD student was selected by the research team as the most suitable candidate for the role, and her experience complemented the rest of the team’s expertise and knowledge. The primary researcher worked with the original Danish team and the principal investigator, who is also a music therapist, to ensure that the training covered all the appropriate areas of music therapy. Before the delivery of the training to any care homes, the primary facilitators ran the training with 2 music therapists, the principal investigator, and second PAMI facilitator, who provided feedback on the content and delivery of the training. The feedback was used to make final changes to intervention before its use in the study. A 3-hour group interactive webinar will be delivered via Microsoft Teams at the study’s beginning. The training is organized at the convenience of the care homes and care staff. The training session follows the manual layout, with staff completing the reflective exercises in the manual during the session. Different learning elements have been used, including written text, pictures, talking, interactive activities, videos, and the music therapist experience, to make the training accessible to many learning styles. During the session, staff are provided with examples for implementing the skills and theories into practice. As PAMI’s central ethos is person centered, what, when, and how frequent skills are used are at the staff’s discretion. Staff are instructed to use PAMI in relation to their resident’s needs, impairments, and preferences. However, they are advised to use PAMI regularly on every shift.

#### Reflective Sessions

BS, a music therapist on the researcher team, will facilitate the reflective sessions with assistance from the primary researcher. The sessions will be conducted once a fortnight for 40 minutes via Microsoft Teams. The sessions hold two purposes: (1) to get staff to reflect on their practices and their experience implementing PAMI and (2) to collect data on using PAMI in care homes. Unlike the training, which has more structure, the reflective sessions are less formal, with sessions guided by the care staff. Three reflective sessions are more structured, each focusing on one of the specific PAMI elements to ensure that staff have understood each element and attempted to implement the skills in the care home. A reflective session outline has been developed by BS to guide the sessions and provides potential questions that the facilitator may wish to ask to prompt discussion.

### Intervention Development

**Figure 3:**
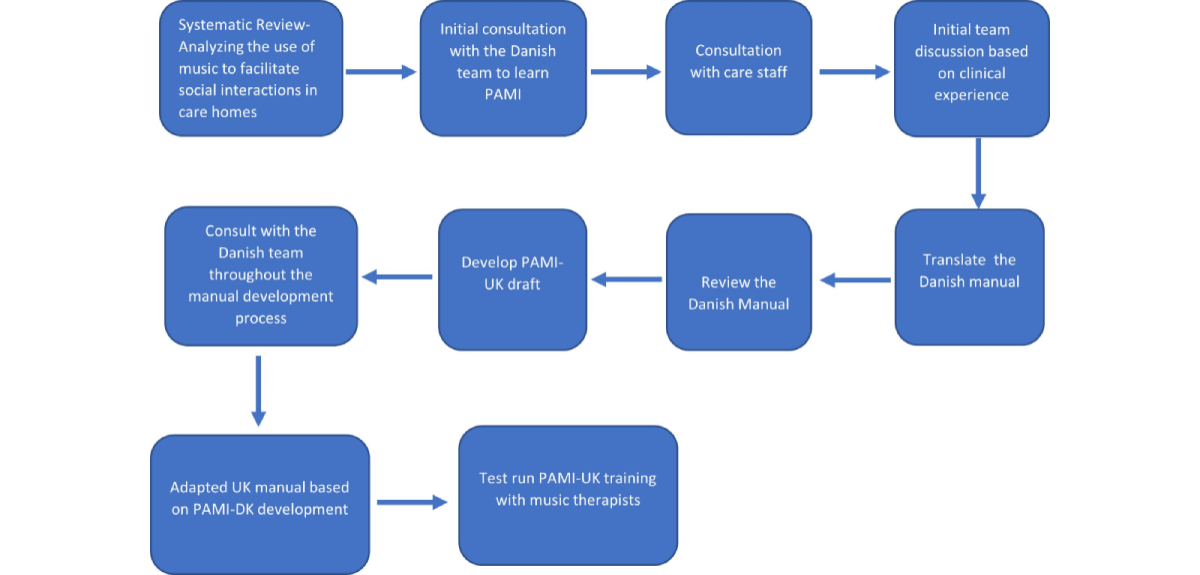
The manual development process for culturally translating and modifying the Person Attuned Musical Interactions (PAMI) intervention for UK care homes. PAMI: Person Attuned Musical Interactions.

The research team completed a multistage process to culturally translate and adapt the Danish PAMI intervention for UK care homes (Figure 3). Throughout the process, the research team liaised with the Danish PAMI team to ensure that any adaptation and modification remained consistent with the PAMI ethos. The research team also incorporated public and patient involvement throughout the development stage to ensure that the intervention was appropriate and suitable for the intended setting and audience. A detailed manual development process is discussed in the primary author’s thesis (Waters, B, Orrell, M, and McDermott, O, unpublished data, March 2023).

During the manual development process, several changes were highlighted to make the intervention more culturally appropriate for UK care homes Table 1.

**Table 1:** The changes between the Danish and Modified Person Attuned Musical Interactions (PAMI).

Element	PAMI^a^	PAMI-M^b^
Information booklets	Theory book Practical workbook for staff Training book for music therapists PowerPoint (Microsoft Corp) for workshop	Training manual containing theory and practical exercises PowerPoint for workshop
Facilitator	PAMI team trains music therapists, who then train care staff in care homes	PAMI team trains care staff in care homes
Training format	In person	Web based
PAMI elements	Ramme (framing in Danish) Regulering (regulation in Danish) Relation (relation in Danish)	Framing Balancing Connecting
Course layout	4 modules Theory and exercise session Practical hands-on training individually and in groups	4 modules Interactive webinar consisting of theory and exercises Reflective sessions
Training length	16 hours over 10 weeks: 8 hours of theory and exercises 4 hours of individual practical training 4 hours of group practical training	3-hour interactive webinar Fortnightly 40-minute reflective sessions
Wording	Some of the wordings did not translate exactly or had different connotations in the United Kingdom: *Svag* (weak in Danish) *Spinkel* (flimsy in Danish)	Weak was changed to soft Flimsy was changed to delicate
Song	Uses Danish well-known songs and folk songs	Uses English well-known songs

^a^PAMI: Person Attuned Musical Interactions.

^b^PAMI-M: Person Attuned Musical Interactions Modified.

### Phase 1: Manual Field-testing Study

#### Overview

Phase 1 aims to review the culturally adapted manual with the intended stakeholders (care staff and residents with dementia). The study will explore the adapted manuals’ suitability, layout, clinical relevance, and culturally adapted appropriateness. Data will also be collected on the factors influencing the implementation of PAMI to determine optimal use, use behavior, and the experience of staff and residents. The data collected in phase 1 will aid revisions to the manual and study design.

Phase 1 is a qualitative field-testing study consisting of interviews, reflective session transcripts, diary entries, and residents’ postintervention questionnaires.

#### Participants

The study will recruit 9 care staff and 9 residents from 3 Lincolnshire care homes. Each staff member will be paired with a participating resident from their care home. Care staff and residents are required to meet the eligibility criteria.

The care staff inclusion criteria are as follows:

- Should be aged >18 years
- Should have the capacity to consent in accordance with the Mental Capacity Act 2005
- Should be permanently employed at the participating care home
- Should have been employed for at least 6 months with plans to remain working at the care home for the study duration (to ensure that staff are familiar with the residents)
- Should work a minimum of 2 shifts a week to ensure sufficient time to implement PAMI into routines
- Should have the ability to read, write, and communicate fluently in English
- Although any member of staff who has direct daily contact with residents can use PAMI, for this study, only staff who assist with activities of daily living or activity staff are eligible
- Should not be currently taking part in any other psychosocial interventions

The care staff exclusion criteria are as follows:

- Staff who are not an employee of the participating care home (eg, agency staff and external health care professionals)
- Members of staff who do not have sufficient direct contact with residents daily (eg, catering or housekeeping)

The resident inclusion criteria are as follows:

- Should be aged >18 years (no upper age limit)
- Should have a diagnosis of dementia as described by the International Classification of Diseases, 11th Revision
- Should have the capacity to consent in accordance with the Mental Health Capacity Act 2005 or have a consultee who could advise on the individual’s wishes if the individual is deemed to lack capacity
- Should have lived at the participating care home for at least 6 months with plans to remain residing at the care home for the duration of the study (to ensure that residents are settled and familiar with the staff)
- Should have sufficient hearing or use hearing aids
- Should have regular interactions with a participating staff member; this will be the staff member they are paired with
- Should not be currently taking part in any other psychosocial intervention research

The resident exclusion criterion is as follows: residents who are residing at the care home on respite or short-term stay.

#### Procedure

The study will run for approximately 10 weeks in each care home, with PAMI being implemented for 8 weeks. The in-reach practitioner will advertise the study to care homes in the in-reach scheme. The manager of eligible care homes will receive the PAMI welcome pack containing welcome letters for staff and residents, which they will be asked to distribute. Interested staff and residents can express their interest via email or to the in-reach practitioner when she attends the care home, after which they will be provided with a participant information sheet and consent form. A consultee will be appointed and provided with information sheets and declaration forms for residents deemed to lack capacity. Consent or consultee declaration forms will be signed for each participant before the start of the study. Once participants have been recruited, staff and residents will be paired into dyads.

At the beginning of the study, staff will receive a paper copy of the PAMI manual, staff and resident demographic questionnaires, a diary entry form, and the Dementia Severity Rating Scale (DSRS) and be asked to complete the questionnaires before the training. A dementia-friendly demographic questionnaire is also provided to assist residents who may struggle with the demographic questionnaire. Each care home is sent a care home demographic questionnaire to complete. The researchers will work with the care home and staff to organize a convenient time to complete the training. After the training, staff will implement PAMI in routines for 8 weeks. Staff are instructed to use the skills in relation to their resident’s needs, impairments, and preferences; therefore, the skills used and frequency of use will vary among staff. However, staff are advised to use PAMI regularly on each shift. Staff are asked to complete the diary form each time they use PAMI with their resident. Once a fortnight, staff will attend a 40-minute reflective session via Microsoft Teams. The facilitators will monitor the study procedure during each reflective session to ensure that no issues or distress have occurred and that both staff and residents are happy to continue. Staff will complete a total of 3 reflective sessions. After the 8 weeks, staff will be invited to a semistructured interview to discuss their experience. The interview will be completed via Microsoft Teams and last approximately 30 minutes. A postintervention resident questionnaire will be sent to staff to complete with their resident if the resident has the ability to communicate their experience. Each care home will receive a participation certificate, and staff members will receive a certificate for completing the PAMI training. At the end of the study, the researcher team will meet to discuss the feedback from participants on the intervention and manual development. Once the team has decided on the required changes, the primary researcher will make revisions to the PAMI manual. The team will also discuss any changes needed to the study design for phase 2; after changes are made, the study will be resubmitted to the ethics committee for approval.

### Phase 2: Manual Evaluation Study

#### Overview

The data collected in the manual evaluation study will aid final manual revisions and aid preparations for future studies. This phase aims to continue to review the culturally adapted manual with the intended stakeholders (care staff and residents with dementia). In addition, preliminary data will be collected on the impact of PAMI on residents and care staff. This phase will be the early stages of the feasibility and piloting stage of the MRC guidelines.

Phase 2 is a mixed methods exploratory study consisting of interviews, reflective session transcripts, diary entries, resident postintervention questionnaire, and pre-post outcome questionnaires.

#### Participants

The study will recruit 25 care staff and 25 residents from 5 Lincolnshire care homes. The care homes will be different from those recruited in the first phase. Each staff member will be paired with a participating resident from their care home. Care staff and residents will be required to meet the eligibility criteria. The same eligibility criteria as those used in phase 1 will be used.

#### Procedure

The study will run for approximately 20 weeks in each care home, with PAMI being implemented for 18 weeks. The recruitment procedure will remain the same as that in phase 1.

At the beginning of the study, staff will receive a paper copy of the PAMI manual, care staff and resident demographic questionnaires, a diary entry form, and the DSRS [57]. A dementia-friendly demographic questionnaire will also be provided to assist residents who may struggle with the demographic questionnaire. Staff will also receive the baseline questionnaires, including the Music in Dementia Assessment Scale (MIDAS) [58], Staff Competence in Dementia scale (SCIDS) [59], QUALIDEM [60], and Modified Nursing Care Assessment Scale (M-NCAS) [61]. Additionally, 8 MIDAS and 8 reflective session prompt sheets will be included in the pack, which staff will require for each reflective session. Staff will be asked to complete the questionnaires before training except for the MIDAS, which staff will be asked to bring to the training. Each care home will be sent a care home demographic questionnaire to complete.

The researchers will work with the care homes and staff to organize a convenient time to complete the training. At the end of the training, staff will be asked to complete the preintervention MIDAS form. The facilitators will then explain that staff should complete 1 reflective session prompt sheet each fortnight before attending the next session and bring the subsequent MIDAS to the session. After the training, staff will implement PAMI in routines for 18 weeks. Staff will be asked to complete the diary form each time they use PAMI with their resident. Once a fortnight, staff will attend a 40-minute reflective session via Microsoft Teams. The facilitators will monitor the study procedure during the session to ensure that there are no issues or distress and that both staff and residents are happy to continue. Staff will complete a total of 8 reflective sessions. During each session, the staff will be asked to complete 1 MIDAS and to use their reflective session prompt sheet to set a goal for the coming fortnight. After 18 weeks, the postintervention questionnaires, the SCIDS [59], M-NCAS [61], and QUALIDEM [60], will be sent to staff to be completed again. At the end of the study, staff will be invited to a semistructured interview to discuss their experience. The interview will be completed via Microsoft Teams and last approximately 30 minutes. A postintervention resident questionnaire will be sent to staff to complete with their resident if the resident has the ability to communicate their experience. Each care home will receive a participation certificate, and staff members will receive a certificate for completing the PAMI training.

#### Data Collection

Data is collected in several ways across the 2 phases. The qualitative data collection methods are the same in both phases. The preintervention and postintervention quantitative questionnaires will be collected only during the manual evaluation phase .

#### Demographic Questionnaires

Staff will be required to complete resident and staff demographic questionnaires. Staff should work with their resident to complete the resident version. Resident’s involvement will be dependent on their impairments and communication abilities. A dementia-friendly questionnaire has been created to allow residents to have more involvement in the research without their impairments hindering participation. However, staff may need to complete the resident questionnaire. The staff questionnaire collects the following details: age, gender, nationality, length of time at the care home, length of time in dementia care, and views on music in dementia care. The residents’ questionnaire collects the following details: age, gender, nationality, length of time at the care home, communication ability, and interest in music.

Participating care homes will complete a demographic questionnaire on the care home. The care home questionnaire collects the following details: care home type, resident type, number of residents in the care home, staff to resident ratio, the current availability of music and activities in the care home, and the availability of Wi-Fi and technology. The question about the availability of Wi-Fi and technology is to ensure that care homes have adequate accessibility for the staff to access the web-based training.

Staff members will complete the DSRS [57] for their residents to determine dementia severity. The DSRS is an informant-based multiple-choice assessment tool. The scale consists of 11 items covering a range of functions impacted by dementia. The areas covered in the DSRS are memory, language, recognition of family members, orientation to time, orientation to place, ability to make decisions, social and community activities, home activities and responsibilities, cleanliness, eating, control of urination and bowels, and ability to get from place to place. A score of 0 to 18 indicates normal to mild dementia, a score of 19 to 36 indicates moderate dementia, and a score of 37 to 54 indicates severe dementia. The DSRS has been calculated to have high internal consistency, high concurrent validity, and high test-retest and high interrater reliability, with carers rating similar to trained clinicians and nonmedical professionals [57]. The DSRS was selected because it is easy to use, can be completed without a researcher, and has high validity and reliability.

#### Diary Entries

Staff will keep a diary throughout the study to document their use of PAMI and thoughts. The primary researcher produced the diary forms. The paper and pencil method was selected over a more technology-based method to ensure that all staff could complete the diary entries during their shift, as the paper and pencil method does not rely upon regular access to a computer, a phone, or other devices. It is also suggested as one of the easiest diary methods; therefore, little instructions are required on it at the beginning of the study [62]. This study will use an event-contingent diary format where staff document every time PAMI interactions occur during the study. The use of diary entries in the PAMI studies allows the researchers to examine the regular use of PAMI in care homes. Data are collected on the time and date of interaction, interaction length time, which PAMI section the interaction corresponded to, what the interaction entailed, the resident’s reaction, PAMI’s impact on the interaction, and space for notes. The diary entry form has been kept simple and made such that it is quick to complete, with an entry taking participants only a few minutes, to improve the completion rate.

#### Reflective Sessions

Although the reflective sessions contribute to part of the PAMI training, they are also a form of data collection. The reflective sessions aim to collect qualitative data on the staff’s experience implementing PAMI in care homes and the impact of PAMI on residents and staff. The reflective sessions are semistructured, with the staff’s experience guiding sessions; therefore, there is no prior expectation of collecting specific information. However, in general, information on the staff’s experience of using the skills, issues concerning the implementation of the intervention, the usability of the manual, and issues with specific skills and case anecdotes of specific interactions will be collected. At the beginning of each reflective session, staff will be asked for permission to audio record the session.

#### Interviews

Staff will be invited to attend an interview at the end of the study to discuss their experience with PAMI. The interviews will follow a semistructured format with a topic guide, but interviewers will ask additional questions based on participants’ responses. Either BW or BS will facilitate the interviews via Microsoft Teams. At the beginning, participants will be asked to provide permission to audio record the interview. Staff will be reminded to use participants’ identity codes when discussing specific residents. However, any information identifying the care home, staff, or residents will be removed and anonymized at the transcribing level. Although face-to-face interviews are considered the gold standard of interview data collection methods [58], all interviews will be completed remotely owing to the uncertainty of COVID-19 restrictions when designing the study. Using a videoconference call to conduct the interviews was selected, as this allows for the closest simulation of an in-person interview [63]. It has also been suggested that when in-person interviews are unavailable, video interviews can create a closer bond between the interviewer and interviewee [63].

#### Quantitative Outcome Measures

A modified version of the MIDAS [58] was selected to measure residents’ music engagement. Typically, the measure is proxy reported, completed by care staff and the music therapist before the beginning of music therapy and then again after the music therapy session to examine the change observed during the session. In the PAMI study, the MIDAS is still proxy reported. However, it will be completed only by care staff at 9 time points at fortnightly intervals, first in training and then at each reflective session. The measure uses a Visual Analogue Scale (VAS) without anchor points, with each VAS consisting of a 100-mm line. Staff will be asked to rate the individual based on their current stage of life; therefore, “highest” means the optimal level that the individual can achieve at that stage of their life. Each VAS has a maximum score of 100, and the MIDAS has an overall maximum score of 500. The MIDAS form consists of 5 VAS on interest, response, imitation, involvement, and enjoyment; a checklist question on major reactions; and any comments or questions. This measure was selected for the PAMI study, as it collects observational data on music engagement using proxy reporting methods, making it suitable for participants with moderate to advanced dementia. Studies investigating the psychometric properties of the MIDAS reported high internal consistency between the 5 VAS items, acceptable test-retest reliability, acceptable concurrent validity, and high construct validity.

The QUALIDEM [60] was selected to measure residents’ quality of life. The QUALIDEM is a dementia-specific proxy rating scale for measuring the quality of life. The scale has been designed to be suitable for use in care homes. The QUALIDEM is completed before and after intervention, with 18 weeks between the 2 points. It is a 37-item multidimensional scale that consists of 9 homogeneous subscales. Each question has a 4-point scoring system ranging from 0 to 3. The scores on the subscales are calculated by adding up the item scores. The higher the score, the better the quality of life that the individual is expected to have for that quality of life domain. The QULIDEM was selected because it measures the emotional and social domains and investigates the care relationship and coping with the nursing home environment, a domain not investigated in other scales. The QUALIDEM has been reported to have moderate interrater reliability; moderate internal structure; and high convergent, discriminate, and concurrent validity [60].

The SCIDS [59] was selected to measure care staff’s competence before and after PAMI. The scale can investigate the effects of training on staff and how these relate to care behavior and the residents’ quality of life. The SCIDS is a professional caregiver’s self-report measure comprising 17 items categorized into 4 subscales: professionalism, sustaining personhood, building relationships, and care challenges. Each question has a 4-point answer scale ranging from 1 to 4. Overall scores range from 17 to 68, with a higher score indicating a higher level of sense of competence. The SCIDS has been reported to have good internal consistency and substantial to moderate test-retest reliability.

The M-NCAS [61] was selected to measure staff burden before and after PAMI. The M-NCAS not only measures behavior but also addresses other aspects of nursing, including staff’s perception of the meaningfulness of residents’ lives and residents’ gratefulness for care. The M-NCAS is a self-reporting staff measure consisting of 32 items. Each item has 2 domains: one explores the occurrence and intensity of the behavior, whereas the other explores the staff’s rating of the difficulty of coping with the behavior. Each domain is scored on a 4-point scale. A lower score on both domains indicates a lower score of staff burden. The M-NCAS has been reported to have excellent to good internal consistency and reliability and moderate construct validity.

### Analysis

#### Demographic Statistics

Data collected from the resident, staff, and care home demographic questionnaires and residents’ DSRS scores will be entered into SPSS Statistics (IBM Corp). The mean and SD will be created for the demographic data.

#### Quantitative Outcome Measures

The data collected from the MIDAS, SCIDS, QUALIDEM, and M-NCAS will be imported into IBM SPSS Statistics. Two-tailed *t* tests will be conducted from the questionnaire data to determine a change before and after PAMI.

#### Qualitative Data

##### Transcribing

Before analyzing the reflective sessions and interviews, audio recordings will be transcribed. The University of Nottingham Automatic Transcribing Service will be used for the first transcribing stage. The service is secure, meets General Data Protection Regulation guidelines, and has an accuracy rate of 70% to 99% [64]. For recordings to be transcribed using the service, they needed to be in the format Mono MP3. The software Audacity (The Audacity Team) will be used to convert the recordings into the appropriate format [65]. Two reviewers will independently review the transcripts to determine accuracy. During transcribing, the names and personal information identifying the residents, staff, or care homes will be removed.

##### Thematic Analysis

NVivo (QSR International) will be used to analyze the transcripts [66]. Due to its flexibility, the researchers will use thematic analysis to analyze the qualitative data. Thematic analysis entails identifying, analyzing, and reporting themes within the chosen set of data [67]. A theoretical approach will be taken, where themes will be generated based on the research aims and questions set at the beginning of the study. Braun and Clark’s [67] 6 steps for thematic analysis will be followed.

Braun and Clark’s [67] 6 steps are as follows: familiarizing yourself with the data, generating initial codes, searching for themes, reviewing themes, defining and naming themes, and producing the report.

The 2 PAMI facilitators who conduct the interviews will also transcribe and conduct the thematic analysis, allowing the researchers to immerse themselves in the data from the beginning. The reviewers will independently generate codes before they are combined and analyzed for inter-reviewer reliability. The 2 reviewers will discuss the generated codes and resolve discrepancies. The reviewers will then independently cluster relatable codes to generate themes before they work together through discussions to develop one set of themes representing the data. Once the 2 reviewers agree, the set of themes will be shared with the research team for feedback.

The qualitative and quantitative data will not be combined in the analysis. The quantitative data are for exploring preliminary statistical findings and testing the appropriateness of the outcome measures for future studies, whereas the qualitative data are for exploring the participants’ experience of using PAMI and reviewing the culturally adapted appropriateness of the manual.

## Results

This project was funded by The Music Therapy Charity in October 2019 as part of a PhD studentship. The study’s first phase began recruitment in September 2021 and continued until January 2022. A total of 8 care staff and 8 residents were recruited, with 5 dyads completing the study. The data have been analyzed that will be used in the primary researcher’s thesis (Waters, B, Orrell, M, and McDermott, O, unpublished data, March 2023) and several papers in peer-reviewed journals. The research team aims to publish the results from the first phase in July to September 2023.

The second phase began recruitment in April 2022 and continued until December 2022. Overall, 20 care staff and 19 residents were recruited with 11 dyads completing the study. The researchers are currently in the stage of analyzing the data that will be used in the primary researcher’s thesis (Waters, B, Orrell, M, and McDermott, O, unpublished data, March 2023) and published in peer-reviewed journals. The research team aims to publish the results from the second phase in October to December 2023.

Dissemination will also occur through conferences. Participating care homes will receive a summary of the results. A summary will be provided to the Lincolnshire NHS trust, which they can use to disseminate the results.

## Discussion

### Anticipated Findings

This study will be the first to investigate the modified PAMI-M. Therefore, the data collected in phases 1 and 2 will provide feedback on the appropriateness of the modified manual for care staff working in UK care homes. The data collected will aid final manual revisions. Due to COVID-19, the intervention was converted from an in-person training tool to an intervention delivered remotely. Therefore, this study will provide a greater understanding of the plausibility of providing PAMI remotely. The study investigates the implementation of PAMI in a real-life setting, highlighting potential barriers and allowing the researchers to adapt the intervention to accommodate.

Previous research has highlighted that implementing meaningful interactions and staff-led music interventions in care homes are challenging because of limited staff time, limited resources, and the lack of knowledge [68,69]. Music therapy skill-sharing can provide staff with music therapy skills to implement in routines without them having to spend additional time or bear additional burden [46,70]. Implementing music interventions in other care tasks can improve task efficiency and reduce staff time. Music therapy skill-sharing is only accessible to a few care homes owing to music therapists’ availability and finances. A music therapy skills-sharing manual such as PAMI will make the intervention accessible to a larger population of care homes. Implementing PAMI in care homes in Denmark has begun and is displaying positive results [48]. This study will investigate whether the modified version of PAMI can benefit residents, care staff, and care homes in the United Kingdom.

### Strength and Limitations

The main strength is the remote element. The remote-based conversion of PAMI has allowed the researchers to continue developing the intervention during the pandemic when there were uncertain COVID-19 regulations. Even as the regulations are dropped, care homes are still battling with care home lockdowns when outbreaks occur; and remote training will allow staff to continue training during these periods. Thinking long term, converting PAMI to remote training would make the intervention more accessible to a wider population of care homes, as the facilitators would not be limited by geographic distance or location. Although the remote element of PAMI is its main strength, it is also the main limitation. All study elements will occur via email and Microsoft Teams; therefore, data collection and implementation will rely on self-report. It will be challenging for the researchers to determine whether staff are using PAMI and are successfully implementing the skills.

### Conclusions

The study will be the first to investigate the modified PAMI. The study will provide feedback on the appropriateness of the manual and explore participants’ experience of using a modified version of PAMI. PAMI has the potential to offer music therapy skill-sharing training to a larger proportion of care homes, ensuring that high-quality appropriate music interventions are provided to residents in care homes where music therapy may be inaccessible. The intervention aims to promote 2-way attuned musical interactions between staff and residents with dementia through musical skills and nonverbal communication and has the potential to benefit residents, staff, and the overall care home.

## Abbreviations

DSRS: Dementia Severity Rating Scale
ENRICH: Enabling Research in Care Homes
MIDAS: Music in Dementia Assessment Scale
M-NCAS: Modified Nursing Care Assessment Scale
MRC: Medical Research Council
MTC: music therapeutic care
NHS: National Health Service
NIHR: National Institute for Health Research
PAMI: Person Attuned Musical Interactions
PAMI-M: Person Attuned Musical Interactions–Modified
SCIDS: Staff Competence in Dementia scale
VAS: Visual Analogue Scale

## Data Availability

Data sharing is not applicable to this study as no data sets were generated or analyzed during the study.
