# Correction: The Development of a UK Culturally Adapted and Modified Version of the Person Attuned Musical Interactions Manual: Protocol for a 2-Phase Mixed Methods Study

**DOI:** 10.2196/56085

**Published:** 2024-01-26

**Authors:** Bryony Waters, Martin Orrell, Orii McDermott

**Affiliations:** 1 Institute of Mental Health School of Medicine University of Nottingham Nottingham United Kingdom

In “The Development of a UK Culturally Adapted Version of the Person Attuned Musical Interactions Manual: Protocol for a 2-Phase Mixed Methods Study” (JMIR Res Protoc 2023; 12(1):e43408) the authors made some modifications.

1. The title “The Development of a UK Culturally Adapted Version of the Person Attuned Musical Interactions Manual: Protocol for a 2-Phase Mixed Methods Study” has been revised to:

The Development of a UK Culturally Adapted and Modified Version of the Person Attuned Musical Interactions Manual: Protocol for a 2-Phase Mixed Methods Study

2. *PAMI-United Kingdom* was revised to *PAMI-Modified*, with all subsequent mentions in the paper being changed from *PAMI-UK* to *PAMI-M*.

3. In Table 1, all instances of the word *Dutch* were revised to *Danish*, as the language of the intervention is Danish.

4. Also in Table 1, the phrases in the title and footnote *PAMI-DK* and *PAMI-DK- Person Attuned Musical Interaction Danish Version* have been replaced by *PAMI* and *PAMI-Person Attuned Musical Interactions*, respectively.

5. In the Corresponding Author's address, *Truiumph Road* was corrected to *Triumph Road*.

6. Some sentences were revised to add the words *modified*, *modification* or *modifying* where necessary, as follows:

In the Abstract, in the section Background, “...a team of researchers in the United Kingdom have culturally adapted the tool.” has been edited to:

...a team of researchers in the United Kingdom have modified and culturally adapted the tool.

In the Abstract, in the section Objective, “This study aims to investigate the appropriateness of the adapted UK manual for UK care homes and to explore...” has been edited to:

This study aims to investigate the appropriateness of the adapted and modified manual for UK care homes and to explore...

In the Abstract, in the section Conclusions, “This study will be the first to investigate the culturally adapted UK PAMI” has been edited to:

This study will be the first to investigate the modified version of PAMI.

In Background, in the section Person Attuned Musical Interactions Manual, “a UK version of PAMI was developed by a team of researchers at the University of Nottingham” has been edited to:

A modified version of PAMI was developed by a team of researchers at the University of Nottingham.

In Background, in the section Person Attuned Musical Interactions Manual, “The first author’s thesis reports the manual development process and the differences between the Danish and UK version of PAMI” has been edited to:

The first author’s thesis reports the manual development process and the differences between the Danish and Modified version of PAMI.

In Methods, under the section Training Session, “BW was the researcher who developed the translated PAMI manual as part of her PhD” has been edited to:

BW was the researcher who developed the modified PAMI manual as part of her PhD.

In Methods, under the section Intervention Development, “...the research team liaised with the Danish PAMI team to ensure that any adaptation remained consistent with the PAMI ethos” has been edited to:

...the research team liaised with the Danish PAMI team to ensure that any adaptation and modification remained consistent with the PAMI ethos.

In Discussion, under the section Anticipated Findings, “This study will be the first to investigate the culturally adapted PAMI. Therefore, the data collected in phases 1 and 2 will provide feedback on the appropriateness of the manual for staff...” has been edited to:

This study will be the first to investigate the modified PAMI-M. Therefore, the data collected in phases 1 and 2 will provide feedback on the appropriateness of the modified manual for care staff...

In Discussion, under the section Anticipated Findings, “This study will investigate whether the UK-adapted version of PAMI can benefit residents,...” has been edited to:

This study will investigate whether the modified version of PAMI can benefit residents,...

In Conclusions, the phrase “The study will be the first to investigate the culturally adapted UK PAMI” has been edited to

The study will be the first to investigate the modified PAMI.

In Conclusions, “...and explore participants’ experience of using PAMI” has been edited to:

...and explore participants’ experience of using a modified version of PAMI.

In the Caption of Figure 2, “PAMI: Person Attuned Musical Interactions” has been edited to:

PAMI-M: Person Attuned Musical Interactions- Modified.

In the caption of Figure 3, the phrase “The manual development process for culturally translating and adapting the Danish Person Attuned Musical Interactions (PAMI) intervention for UK care homes. PAMI-DK: Person Attuned Musical Interactions Danish version; PAMI-UK: Person Attuned Musical Interactions United Kingdom” has been edited to:

The manual development process for culturally translating and modifying the Person Attuned Musical Interactions (PAMI) intervention for UK care homes. PAMI: Person Attuned Musical Interactions.

In the caption of Table 1, “The changes between the Danish and UK Person Attuned Musical Interactions (PAMI)” has been edited to:

The changes between the Danish and Modified Person Attuned Musical Interactions (PAMI).

The correction will appear in the online version of the paper on the JMIR Publications website on January 26, 2024, together with the publication of this correction notice. Because this was made after submission to PubMed, PubMed Central, and other full-text repositories, the corrected article has also been resubmitted to those repositories.

